# Books Reviewed

**Published:** 1864-10

**Authors:** 


*A Handbook of Uterine Therapeutics, by* Edward John Tilt, M. D., *Member of the Royal College of Physicians; Consulting Physician to the Farringdon General Dispensary; Fellow of the Royal Medical and Chirurgical Society, and of several British and Foreign Societies. New York:* Wm. Wood & Co., 61 *Walker street*, 1864.

This is a work which discusses in a candid and impartial manner the various modes of treating the inflammatory affections of the womb, which have been adopted with the apparent object of deciding the real value of our uterine therapeutical agents.

The introduction contains timely suggestions as to the mode of examining patients, and the character of uterine remedies in general—their indications and value. The first chapter is upon Uterine Dietetics; of stimulants we quote the following from our author: "Few patients consult me who have not at some time or other been drenched with wine, ale or porter, often in direct opposition to their safer instincts. I have seen young ladies rendered hysterical by undetected uterine inflammation, who were kept half drunk, for weeks, on stout or wine. However indispensable in certain forms of fever, large quantities of alcohol are highly injurious in purely inflammatory affections. The worst of this system is, that it panders to the strong propensities of our race; for it is not to be forgotten that we are akin to those nations whose early view of paradise was to drink perennial mead out of their enemies' skulls, and that our grandfathers generally brought festivities to a close *under* the table."

Vaginal injections, irrigations, enemata and their uses, suppositions, etc. are duly considered, and their value we think very correctly estimated.

In antiphlogistic treatment, he speaks of "bleeding as more valuable than fashionable," speaking fully of its abuses and utility, also of leeches, scarifications, purgations, blisters, seatons, issues, counter-irritants, etc. Speaking of mercury as an antiphlogistic we again quote our author; he says, under the heading, "*Alterative and Fluidfiant Medicines*," "Though I believe in the utility of mercury as an antiphlogistic, and as a means of acting on the liver, I quite agree with those who protest against its blind use as still adopted by many in this country; I mean, the plan of giving a *mild* course of *mercury* whenever a case is obscure and protracted. Some of my patients have not yet recovered from the "mild course of mercury" to which they were subjected twenty years ago; and Dr. Wright's analysis have proved how greatly the constituents of the blood can be injured by mercury. * * * * * Those who pursue this mercurial plan of treatment, adopt it in all cases of uterine inflammation in connection with vaginal injections and other judicious measures; and as many cases soon recover, the credit of cure is given to the small doses of mercury, whereas the patients would have recovered just as soon if that remedy had been omitted, provided the rest of the treatment had been carried out." * * * * * "When, however, all remedies have been exhausted, and the patients still continue to suffer from internal metritis, with chronic inflammation of the body of the womb, I think it right to try the effects of mercury pushed to full salivation."

It would be pleasant and profitable to follow our author through the entire volume and review his positions, speaking in detail of them all. He has noticed nearly every remedial measure adapted to uterine disease, and though he has recommended some things which are perhaps of questionable value, and discarded others which are regarded as often necessary and useful, still, the book, as a volume, is quite free from indiscriminate approval of remedies or unfounded rejection of measures which he has not himself proved by experience. The work is exceedingly well considered and well arranged, and the whole subject of uterine therapeutics is very candidly and correctly presented. It is a work which should be carefully read; it will do much to obviate a tendency to routine in practice, and show how mistaken are the views often entertained as to the nature of uterine displacements and the necessity of cure; it will give truthful ideas of nearly all our measures for treating the diseases of women. To the body of the work is appended a very choice and select formulary, with the view to "substitute definite quantities of valuable remedies for uncertain preparations sometimes used, and to suggest inoffensive preparations in the place of some that are needlessly filthy."

---

*Essays on Infant Therapeutics; to which are added Observations on Ergot; History of the origin of the use of Mercury in Inflammatory Complaints; together with the Statistics of the Deaths from Poisoning in New York in the years* 1841–2–3. By John B. Beck, M. D., *Professor of Materia Medica and Medical Jurisprudence in the College of Physicians and Surgeons of the University of the State of New York; Corresponding Member of the Royal Academy of Medicine of Paris; Corresponding Member of the Medical Society of London; one of the Vice Presidents of the Academy of Medicine of New York, etc. Third edition, enlarged and revised.* New York: Wm. Wood & Co., 1864.

This volume contains chapters upon the effects on the young subject of opium, emetics, mercury, blisters and sinapisms, bloodletting, ergot, and an account of the origin of the use of mercury in inflammatory complaints; also on the deaths from poisoning in the city and county of New York during the years 1841–'42 and '43. To this is added an appendix, composed mostly of cases illustrative of the positions contained in the body of the work. The opinions of so distinguished an observer are worthy of the greatest respect, and the little volume shows in every sentence the candor and correctness of a most intelligent and careful obeervdr. Every position taken is sustained by irrisistible argument and illustration, and those who read the book carefully will adopt the views of the author, whatever may be their prejudices; it consists of a collection of facts, united by argument and explanation, in such manner as to be absolutely convincing. Every physician should read *Beck on Infant Therapeutics.*

---

*Transactions of the State Medical Society of Indiana, at the Fourteenth Annual Session, held in the city of Indianapolis May* 18, 1864.

The Indiana State Medical Society assembled in College Hall, Indianapolis, and was called to order by the Vice President, John Moffitt, who also gave the Annual Presidential Address. W. Lockhart, M. D. of Danville, Ind., made report of interesting cases of vaccination. Dr. Hutchinson read a paper upon the fevers of Indiana, which, from cursory examination, we think interesting and valuable. Dr. Booker of Castleton, presented a paper upon camp diarrhœa. Speaking of opiates and astringents he says: "If the Surgeon-General had stopped their indiscriminate use instead of calomel and tartar-emetic, he would have done better." The volume contains code of ethics and other interesting matter which we have not space to notice.

---

BOOKS RECEIVED.

